# Death Associated With Coronavirus (COVID-19) Infection in Individuals With Severe Mental Disorders in Sweden During the Early Months of the Outbreak—An Exploratory Cross-Sectional Analysis of a Population-Based Register Study

**DOI:** 10.3389/fpsyt.2020.609579

**Published:** 2021-01-08

**Authors:** Martin Maripuu, Marie Bendix, Louise Öhlund, Micael Widerström, Ursula Werneke

**Affiliations:** ^1^Division of Psychiatry, Department of Clinical Sciences, Umeå University, Umeå, Sweden; ^2^Department of Clinical Neuroscience, Center for Psychiatry Research & Stockholm Health Care Services, Stockholm County Council, Karolinska Institutet, Stockholm, Sweden; ^3^Division of Psychiatry, Sunderby Research Unit, Department of Clinical Sciences, Umeå University, Umeå, Sweden; ^4^Department of Clinical Microbiology, Umeå University, Umeå, Sweden

**Keywords:** coronavirus, COVID-19, severe mental disorder, death, risk factors, mortality, psychotic disorder, bipolar disorder

## Abstract

**Background:** Individuals with severe mental disorder (SMD) have a higher risk of somatic comorbidity and mortality than the rest of the population. We set up a population-based study to assess whether individuals with SMD had a higher risk of death associated with a COVID-19 infection (COVID-19 associated death) than individuals without SMD.

**Methods:** Exploratory analysis with a cross-sectional design in the framework of a population-based register study covering the entire Swedish population. The Swedish Board for Health and Welfare (Socialstyrelsen) provided anonymized tabulated summary data for further analysis. We compared numbers of COVID-19 associated death in individuals with SMD (cases) and without SMD (controls). We calculated the odds ratio (OR) for the whole sample and by age group and four comorbidities, namely diabetes, cardiovascular disease, hypertension, chronic lung disease.

**Results:** The sample comprised of 7,923,859 individuals, 103,999 with SMD and 7,819,860 controls. There were 130 (0.1%) COVID-19 associated deaths in the SMD group and 4,945 (0.06%) in the control group, corresponding to an OR of 1.98 (CI 1.66-2.35; p < 0.001). The odds were 4-fold for the age groups between 60 and 79 years and 1.5-fold for cardiovascular diseases. Individuals with SMD without any of the risk factors under study had 3-fold odds of COVID-19 associated death.

**Conclusion:** Our preliminary results identify individuals with SMD as a further group at increased risk of COVID-19 associated death. In regard to comorbidities, future studies should explore the potential confounding or mediation role in the relationship between SMD and COVID-19 associated deaths.

## Introduction

Severe mental disorders (SMD), such as bipolar or psychotic disorders are associated with premature mortality. SMD has a substantial impact on life expectancy, which may be shortened by 10 to 20 years (1–3). Somatic disorders account for at least 50% of this shortened life expectancy (3). Cardiovascular conditions are the main cause of premature death (3–5). Infectious diseases also seem to contribute to a shortened life expectancy in individuals with SMD (1, 2). It currently remains unclear whether individuals with severe SMD have an increased risk of death associated with coronavirus SARS-CoV-2 (COVID-19) infection. The question is important, since individuals with SMD may have an excess risk of factors linked to an adverse outcome of COVID-19 infection. Such include comorbidities such as cardiovascular conditions, diabetes, chronic respiratory disease, hypertension and obesity (6, 7). At the same time, individuals with SMD have less access to somatic health care (5, 8). Therefore, there are reasons to believe that individuals with SMDs experience a higher mortality associated with COVID-19 infection than the rest of the population. If this assumption held true, individuals with SMD would join the category of individuals at increased risk of an adverse clinical course of COVID-19 infection, along with people who are older, obese or who have pre-existing somatic conditions. We set up a national register study to examine this hypothesis. In order to be able to make health professionals aware of this potential risk group as soon as possible, we conducted a first exploratory analysis, which we report here.

### Aim

To assess the risk of death associated with COVID-19 infection (COVID-19 associated death) in individuals with SMD. We tested the following hypothesis: Individuals with SMD have a higher risk of COVID-19 associated death than individuals without SMD (reference population).

## Methods

### Study Design

The current study is part of a longitudinal population-based register study based on the Swedish National Patient Register, the Swedish National Death Register, and the Swedish Prescribed Drug Register. All registers are held by the Swedish Board for Health and Welfare (Socialstyrelsen). For this exploratory analysis, we chose a cross-sectional design, linking the Swedish National Patient Register and the Swedish National Death Register. The data manager at the Swedish Board for Health and Welfare linked the respective registers through the unique personal identification number (Swedish personal number). Anonymized tabulated summary data was made available to the research team for further analysis. We checked our whole method against the Strobe checklist (Appendix 1) (9).

### Data Sources

The Swedish National Patient Register is based on diagnoses for both in and outpatient care in specialized medicine (secondary care). Diagnoses from general practitioners (primary care) are not included in this register. The Swedish National Death Register includes all Swedish persons that have died. The cause of death is established in either primary or secondary care, depending on where the death has occurred. The Swedish Prescribed Drug Register contains data on treatments that were dispensed at a pharmacy.

### Sample

We included the whole Swedish population of at least 20 years of age by 31 Dec 2019. We defined individuals with a diagnosis of SMD as cases and all other individuals as controls. As the sample covered the whole adult Swedish population, all individuals fell either into the category “SMD” or the category “without SMD.” Therefore, there were no other exclusion criteria other than age < 20 years. The cut-off of 20 years was chosen, because we used multiples of 10 years to stratify our data. Eighteen to twenty years was left out, because of the short age span and expected low risk of COVID-19 associated death in young people.

### Variable Definitions

#### Outcomes

In Sweden, the first confirmed case of COVID-19 infection was reported on 31st January 2020 (10). The first COVID-19 related death was reported on 11th March 2020 (11). Our outcome was COVID-19 associated death, registered as such by the Swedish Board for Health and Welfare. We included all COVID-19 associated deaths occurring over a 3 month period, from 11 March 2020 until 15 June 2020.

We analyzed COVID-19 associated deaths as a dichotomous yes/no variable. The Swedish Board for Health and Welfare bases the criteria COVID-19 associated death on the underlying cause of death recorded on the death certificates. Two codes of the 10th version of the International Classification of Diseases (ICD-10) (12) are currently used, U07.1 or U07.2. Both codes fall into the ICD-10 chapter for provisional assignment of new diseases of uncertain etiology or emergency use (U00–U49). U07.1 is used when COVID-19 has been confirmed by laboratory testing irrespective of severity of clinical signs or symptoms. U07.2 is used when COVID-19 is diagnosed clinically or epidemiologically, but laboratory testing is inconclusive or not available (12). The World Health Organization (WHO) defines as “epidemiologically linked” “being linked to a cluster with at least one confirmed case” (13).

#### Exposures

The main exposure was SMD. Into the SMD variable, we included psychotic disorders with ICD-10 codes F20, F22, F25, and bipolar disorders/single manic episodes with ICD-10 codes F30, or F31. We combined these disorders into one category to increase the sample size for our relatively rare outcome, death. We focused on psychotic and bipolar disorders, since this allowed comparison with a previous Swedish register study reporting on somatic comorbidities, including lung diseases, and excess mortality in both conditions (1, 2). Individuals were included in the SMD category when there were at least two registered diagnoses between 1998 and 2019.

We also explored four somatic comorbidities, diabetes, cardiovascular diseases, hypertension and chronic respiratory diseases, which were available in the registers. In our choice of comorbidities, we were guided by the Swedish National Board of Health and Welfare, which defined groups deemed to have increased risk for severe course of Covid-19 infection in April 2020. This list was last updated 2 June 2020. Besides age over 70, this list includes cardiovascular disease, hypertension, diabetes, chronic renal disease, chronic respiratory disorders and other somatic disorders (14). From this list, we then chose our four comorbidities for two reasons, (a) being known to be associated with severe mental disorders, and (b) being of sufficient sample size to explore the relatively rare outcome of death. Comorbidities with lover prevalence would lead to only few individuals to be included, in which case the Swedish Board of Health and Social Welfare would withhold much of the information due to confidentiality reasons.

We introduced a fifth category “none of the above,” which included all other individuals. As the use of the term risk factor is ambiguous, we have used the term comorbidities instead, as a personal attribute linked to the (binary) outcome death and assumed to be present at the same time as the outcome or potentially having a role in the aetiological mechanism (15). We checked these comorbidities for a time period from 2015 to 2019, based on diagnosis or pharmacological treatment received, using ICD and Anatomical Therapeutic Chemical Classification System (ATC) codes. We used the following ICD and ATC codes, (a) diabetes ICD E10-14, ATC A10, (b) cardiovascular diseases ICD I20-25, I48, I50, I61, I63, I64.9, I69.1, I69.3, I69.4, I69.8, I70, (c) hypertension ICD I10.9, I11–13, I15, ATC C02, C03, C07AB02, C08CA, C09, and (d) chronic respiratory diseases ICD J40–47, J60–67, J68.4, J70.1, J70.3, J96.1, J96.8, E 84.0. We stratified age into the following groups, 20–39, 40–59, 60–69, 70–79, and 80+ years.

### Statistical Methods

**Figure 1:**
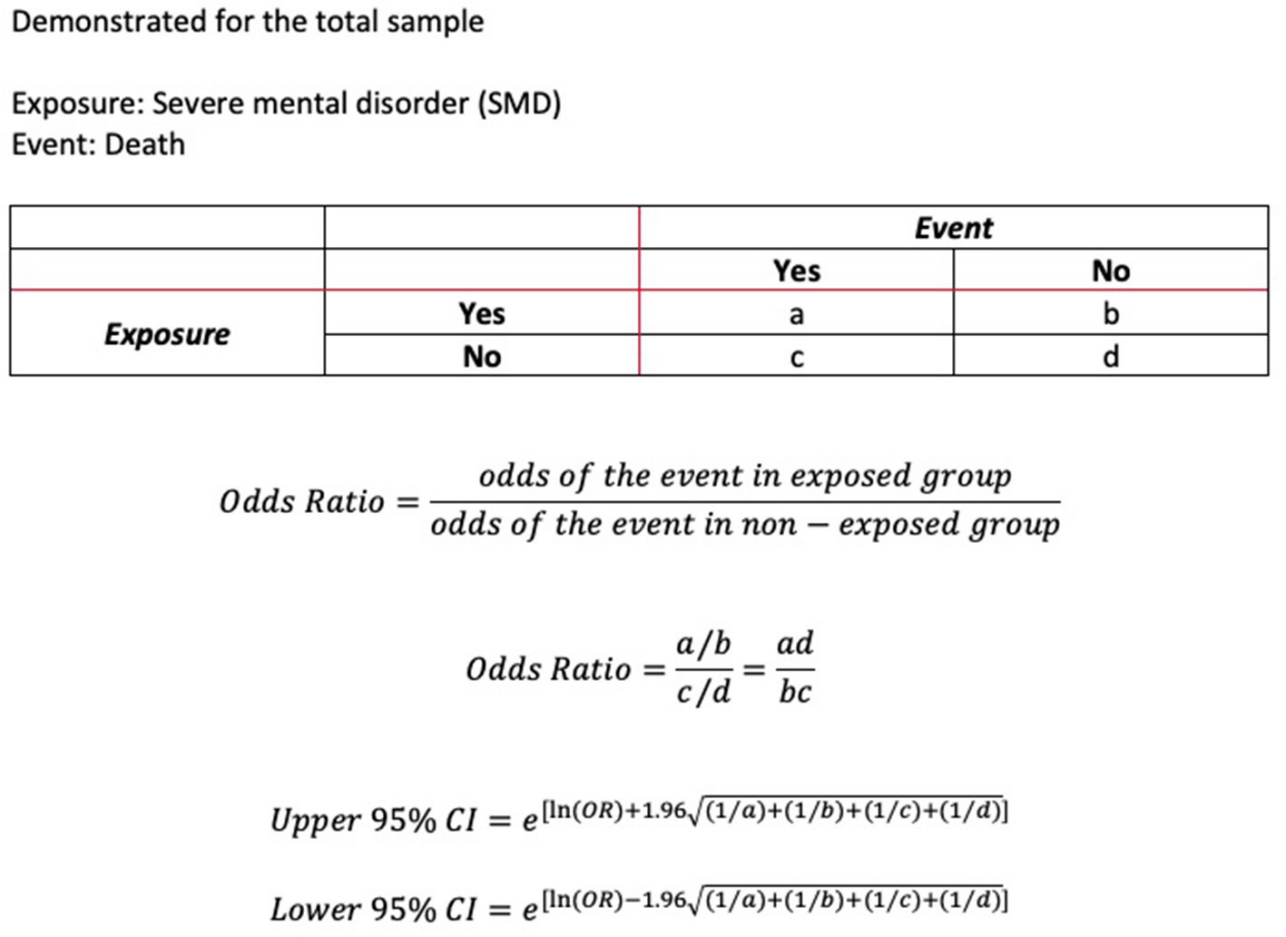
Method for calculation of odds ratios and 95% confidence interval.

The data was provided as summary data in anonymized form by the Swedish Board of Health and Welfare. Statistical analysis underlying this summary data was performed by a statistician at the National Board of Health and Welfare. The research group then analyzed this data further. The data included information stratified according to case and controls on number of individuals in each age group and number of individuals in each comorbidity category. From this data, we calculated the odds ratios (OR) for COVID-19 associated death for (a) the whole sample, (b) for each age group, and (c) for each risk factor category. For OR according to age-group, each death was only counted once. For OR according to comorbidity category, a death could be counted several times when it appeared in more than one comorbidity category. As for this preliminary report we only had access to summary data, we could therefore only stratify by one variable at the time, age or comorbidity but not age and comorbidity. We calculated confidence intervals (CI) using Woolf's formula for the standard error. Significance was tested with z-test. The significance level as a measure of random variability as a source of error (16) was set to 0.05 throughout, corresponding to a 95% CI. As we had to rely on summary data and had no access to individual level data, we constructed the respective formulae in Excel (Figure 1). We then confirmed our analysis using the internet based statistical software MedCalc (17). We also used MedCalc to derive our *p*-values. Where available, we compared the results of our z-test to the results of the Fisher exact test, provided by the National Board of Health and Welfare. Identification of significant relationships was 100% concordant.

#### Missing Data

The number of deaths was available for all age groups. However, for some comorbidity categories, the number of deaths had been withheld due to confidentiality reasons. For summary estimates of comorbidities in the whole age group, we set missing data to 0.

### Ethics and Consent Procedures

The study was approved by the Swedish Ethical Review Authority (DNR 2020-02759) and conducted according to the declaration of Helsinki. The data originated from routine information collected by the Swedish Board of Health and Welfare, then made available as summary data in anonymized form for this first exploratory analysis. As only anonymized data was provided and potentially identifiable data was withheld, informed consent was not required.

## Results

### Baseline Characteristics of the Samples

The sample comprised of 7,923,859 individuals, 103,999 (1.3%) with SMD and 7,819,860 (98.7%) controls. As to be expected with a sample of that size, all differences regarding age groups and comorbidities under study were significant with *p* < 0.001 (Table 1).

**Table 1:** Age and risk factor distribution in patients with severe mental disorders vs. reference population.

	**Population with severe mental disorder**^**a**^ ***n*** **=** **103,999**		**Reference population** ***n*** **=** **7,819,860**	
	***n***	**%**	***n***	**%**
**Age groups**				
20–39	31,246	30.0	2,662,638	34.0
40–59	40,899	39.3	2,555,319	32.7
60–69	17,163	16.5	1,091,275	14.0
70–79	10,986	10.6	978,027	12.5
80+	3,705	3.6	532,601	6.8
**Comorbidities across all age groups**^**b**^^**c**^				
Diabetes	8,012	7.7	327,738	4.2
Cardiovascular disease	7,308	7.0	573,187	7.3
Hypertension	10,993	10.6	779,557	10.0
Chronic lung disease	5,664	5.4	231,686	3.0
None of above	83,044	79.9	6,599,259	84.4

n, number.

a Diagnoses of severe mental disorders (bipolar or psychotic disorder) recorded between 1998 and 2019.

b Diabetes, cardiovascular disease, hypertension, chronic lung disease, recorded between 2015 and 2019.

c Individuals could have more than one risk factor. Hence, the number of risk factors exceeded the number of individuals.

### COVID-19 Associated Death

There were 130 (0.13%) deaths associated with COVID-19 infection in the SMD group and 4,945 (0.06%) in the control group. In the SMD group, 90.0% of COVID-19 diagnoses were ascertained by test (U07.1). In the reference group, 91.2% of COVID-19 diagnoses were ascertained by test (U07.1). There were no significant differences in the proportion of COVID-19 diagnoses ascertained by test (U07.1) in both groups (*p* = 0.419). The SMD group had double odds of COVID-19 associated death (OR 1.98, CI 1.66–2.35; *p* < 0.001). Regarding age, higher odds were found for individuals with SMD in the older age groups, about 4-fold in the age groups of 60–69 years and 70–79 years, and about 2-fold in the age group 80+ years (all *p* < 0.001). For the age group of 40–59 years the OR narrowly missed the pre-defined significance level (*p* = 0.052). For the age group 20–29 years, the OR could not be calculated since data were withheld due to confidentiality reasons. Regarding comorbid conditions in individuals with SMD, 1.5-fold odds were found for cardiovascular diseases (*p* < 0.007). The other three comorbidities, diabetes, hypertension and chronic lung disease did not reach significance in terms of the pre-defined significance level. Individuals with SMD who had none of the sampled comorbid conditions had about 3-fold increased odds of COVID-19 associated death (*p* < 0.001) (Table 2).

**Table 2:** COVID-19 associated death having occurred between 1 January 2020 and 15 June 2020 in patients with severe mental disorder vs. reference population according to age or risk factors.

	**Population with severe mental disorder** ***n*** **=** **103,999**^**a**^		**Reference population** ***n*** **=** **7,819,860**				
	***n***	**%**	***n***	**%**	**OR**	**LCI**	**UCI**
**Total sample**							
N deaths	130	0.13	4,945	0.06	1.98	1.66	2.35
**According to age group [years]**							
20–39	–	–	–	–	– ^b^	–	–
40–59	6	0.01	167	0.01	2.24	0.99	5.07
60–69	21	0.12	296	0.03	4.52	2.90	7.03
70–79	51	0.46	1,094	0.11	4.16	3.14	5.52
80+	52	1.40	3,388	0.64	2.22	1.69	2.93
**According to comorbidity**^**c**^							
Diabetes	32	0.40	1,161	0.35	1.13	0.79	1.60
Cardiovascular disease	46	0.63	2,431	0.42	1.49	1.11	1.99
Hypertension	43	0.39	2,644	0.34	1.15	0.85	1.56
Chronic lung disease	13	0.23	633	0.27	0.84	0.48	1.46
None of above	48	0.06	1,328	0.02	2.87	2.15	3.83

n, number; OR, odds ratio; LCI, lower 95% confidence interval; UCI, upper 95% confidence interval.

a Diagnoses of severe mental disorders (bipolar or psychotic disorder) recorded between 1998 and 2019, risk factors recorded between 2015 and 2019.

b OR not calculated because data withheld due to confidentiality reasons.

c n Deaths counted for each risk factor. i.e., deaths associated with more than one risk factor will appear several times.

## Discussion

### Main Findings

This is one of the first population-based studies reporting on the risk of COVID-19 associated mortality in individuals with SMD during the early months of the coronavirus outbreak. We found that individuals with SMD had almost double odds of COVID-19 associated death compared to the reference population without SMD. Individuals with SMD aged between 60 and 79 years were particularly vulnerable with more than 4-fold odds of COVID-19 associated death. Of the four comorbidities available for study, cardiovascular disease increased the odds of COVID-19 associated death by 50%. Individuals without any of the four comorbidities under study, had 3-fold odds of COVID-19 associated death. Our findings are in line with other recently published population-based studies from the US and the UK. One US study covered health records of 61 million adult patients across the country until 29 July 2020. This study compared patients with a recent diagnosis of mental disorder, including ADHD, bipolar disorder, depression or schizophrenia with all other patients without mental disorder defined in this way. Patients with mental disorder and COVID-19 infection also had a nearly 2-fold increased death rate with 8.5% vs. 4.7% among COVID-19 patients without mental disorder (18). Another analysis from the same database until 15 June 2020 showed that patients with substance use disorder (SUD) and COVID-19 had an approximately 30% increased death rate with 9.6% vs. 6.6% among COVID-19 patients without SUD (19). A UK biobank study explored the association between death, COVID-19 infections with tests performed between 16 March 2020 and 26 April 2020, and pre-existing medical conditions as ascertained between 2006 and 2010. This study included 269,070 COVID-19 positive individuals aged between 65 and 86 years. There were 507 COVID-19 positive inpatients, 141 (27.8%) of whom died. Five pre-existing conditions significantly increased the odds of dying. Dementia increased the odds of dying 7.3-fold, diabetes 3.1-fold, chronic obstructive pulmonary disease (COPD) 1.9-fold, pneumonia 1.9-fold, and depression 1.8-fold (20).

### Severe Mental Disorder and Risk of Death From Other Respiratory Infections

At the time of writing, evidence regarding the association between SMD and COVID-19 infection is only emerging. But our findings are also in line with studies exploring the association between SMD and other (non-COVID-19) respiratory infections. A Swedish register study showed a 3- to 4-fold increased risk of death due to influenza or pneumonia in individuals with bipolar disorder (1). In individuals with schizophrenia, the risk was increased 7-fold (2). An American study also found a 7-fold increased risk of death due to pneumonia or influenza in adults with schizophrenia (21). A Danish register study explored all individuals hospitalized for any infection between 1995 and 2011. Individuals with bipolar or psychotic disorder had 52% increased mortality risk within 30 days after their infection (22). We could not find any study that explicitly explored risk factors associated with death from respiratory infections in individuals with SMD.

### Underlying Medical Conditions Associated With a Severe Outcome From COVID-19 Infection in the General Population

The evidence regarding risk factors for a severe outcome from COVID-19 infection is rapidly evolving. The US Centers for Disease Control and Prevention (CDC) have collated a list of underlying medical conditions that increase a person's risk of severe illness from COVID-19, defined as hospitalization, admission to the ICU, intubation or mechanical ventilation, or death. On this list, these conditions are rated into three categories according to quality of evidence, (a) strongest and most consistent evidence, (b) mixed evidence, and (c) limited evidence. Conditions with the strongest and most consistent evidence include cancer, chronic kidney disease, COPD, heart conditions such as heart failure, coronary artery disease, or cardiomyopathies, obesity with a body mass index (BMI) > 30 kg/m^2^, severe obesity with a body mass index (BMI) > 40 kg/m^2^, sickle cell disease, smoking, solid organ transplantation and type-2 diabetes mellitus. Conditions with mixed evidence include asthma, cerebrovascular disease, hypertension, pregnancy, use of corticosteroids and other immunosuppressive medications. Conditions with limited evidence include bone-marrow transplantation, HIV, immune deficiencies, inherited metabolic disorders, liver disease, neurological conditions specific to pediatric conditions, other chronic lung diseases, overweight with a BMI > 25 but <30 kg/m^2^, complex pediatric conditions, thalassaemia and type-1 diabetes mellitus (23). Notably, SMD is not mentioned in this list.

The CDC list identifies a large number of potentially underlying medical conditions. Most of these could increase mortality risk in their own right. Thus, COVID-19 associated death may not necessarily be caused by COVID-19 infection. CDC statistics show that in just 6% of deaths involving COVID-19 infection, COVID-19 was the only cause mentioned. For the 94% deaths with conditions or causes in addition to COVID-19, there were on average 2.6 additional conditions or causes per death (24). An audit of 122 Covid-19 associated deaths from Östergötland County, Sweden, came to similar results. In only 15% of deaths, COVID-19 infection was given as the direct cause. In 70% COVID-19 infection was thought to be a contributory factor but not the main cause. In the remaining 15%, the death could not be related to COVID-19 infection (25).

### Underlying Medical Conditions Associated With Death From COVID-19 Infection in Individuals With Severe Mental Disorder

In bipolar disorder and schizophrenia, comorbidity with at least one somatic condition is very common (26, 27). When acute physically ill, individuals with SMD may then be sicker. One insurance claims study from Taiwan compared the risk of death in an intensive care unit (ICU) between 203 patients with schizophrenia and 2,036 matched controls. In ICU, patients with schizophrenia were sicker, had a higher risk of acute organ dysfunction and death (28). Therefore, it is plausible that individuals with SMD may have a higher risk of COVID-19 associated death.

For our study, we chose four comorbidities thought to be more prevalent in individuals with SMD (21). We chose these comorbidities during the set-up of the study. At the time, evidence regarding comorbidities and other risk factors was only emerging. Therefore, we made an informed guess that these four risk factors could affect the risk of COVID-19 associated mortality. But only cardiovascular disease led to a significantly increased OR in individuals with SMD. Cardiovascular conditions belong to the CDC category of risk factors with the strongest and most consistent evidence (23). For hypertension and chronic lung disease, we did not find an increased risk of death. Hypertension belongs to the CDC category of risk factors with mixed evidence (23). Chronic lung disease includes conditions that fall in the CDC categories of either mixed or limited evidence (23). Surprisingly, diabetes did not significantly increase the risk of COVID-19 associated death in our study. However, our study is only exploratory, sampling the first 3 months of the outbreak. There is also a chance that diabetes was underestimated in individuals with SMD. Despite rising awareness, diabetes may be one of the comorbidities easily missed in individuals with SMD (29, 30).

Our current analysis is exploratory, covering the first 3 months of the COVID-19 outbreak in Sweden. Therefore, the comorbidity profile may change in future analyses. In our study, the OR was highest for the individuals with SMD who did not have any of our chosen four comorbidities. There is no plausible mechanism which could explain a direct role of SMD in the pathophysiology of COVID-19 infection, which would make SMD a risk factor in its own right. More likely, there were other factors and/or comorbidities, not captured by our study, that increased the mortality risk from COVID-19 infection in individuals with SMD. Yet, this clear excess mortality risk identified makes individuals with SMD a risk group of their own, even if SMD *per se* is not involved in the pathophysiology of COVID-19 infections or its clinical course. As already argued by Wang et al. based on their finding of excess mortality in patients with mental disorder (18), individuals with SMD should be added to the groups already known to be at risk of serious illness from COVID-19 infection, i.e., the elderly, the obese or those with somatic comorbidities. Ultimately, longitudinal studies are required to identify the factors and comorbidities that increase the risk of an adverse outcome from COVID-19 infection including death. This puts clinicians and policy makers at a moral dilemma. In view of the second wave of the pandemic and its threats to human health and lives clinicians and policy makers need to act according to the available evidence even if this evidence is currently incomplete.

### Other Medical Factors and Conditions That May Specifically Associated With Severe Mental Disorder and/or Its Treatment

There may be other factors in individuals with SMD that can increase the risk for COVID-19 associated mortality. Such factors may either be associated with SMD itself, its pharmacological treatment or with a combination of both underlying SMD and its pharmacological treatment.

Factors associated with SMD include smoking and substance use disorder (SUD). Both remain highly prevalent in individuals with SMD (31–33). They increase the risk of pneumonia, cardiovascular disease, and compromised immunity. Increased risk for infection and subsequent worse outcomes may also result from difficulties to adhere to preventive measures (34).

Medical conditions associated with the pharmacological treatment include medication associated pneumonia, neutropenia and QT prolongation. Exposure to first-generation antipsychotics (FGA) or second-generation antipsychotics (SGA) may double the risk of pneumonia (35). Mortality from pneumonia may also increase (36). Mood stabilizers such as valproate and carbamazepine may be risk neutral. Lithium may be protective (37, 38), for reasons yet to be explained. Benzodiazepines and benzodiazepine related drugs (BZRD), taken by 30 to 60% of individuals with schizophrenia or bipolar disorder, are other concerns (39–42). Neutropenia and its extreme form agranulocytosis can occur with a variety of antipsychotics and mood-stabilizers, particularly with clozapine and carbamazepine (43). Some of the agents used to treat COVID-19 infection such as chloroquine, hydroxychloroquine, azithromycin, and lopinavir can also cause neutropenia (44). Hence, individuals with SMD taking such agents need careful monitoring (45). QT prolongation is a potentially dangerous adverse effect increasing the risk of torsade de pointes and current cardiac death. Many antipsychotics can prolong QT interval. Citalopram, escitalopram, tricyclic antidepressants and methadone can also prolong the QT interval. Intravenous administration, combination therapy or excess doses also increase the risk for QT prolongation (43). These psychotropic agents may become problematic in combination with somatic drugs also increasing QT interval, used for treating a COVID-19 infection. The latter group includes some antibiotics and antiarrhythmics, chloroquine, hydroxychloroquine and the antiviral and histamin-2 antagonist famotidine (44). It currently remains unclear how often such interactions with psychotropic drugs occur in the context of COVID-19 treatment.

Medical conditions associated with the underlying SMD and its treatment include obesity and venous thromboembolism (VTE). The likelihood of obesity is 2.8 to 4.4 times increased in individuals with schizophrenia and about 1.2 to 1.7 times increased in individuals with bipolar disorder or major depression (46). In part, this increased risk is associated with psychotropic medications. Antipsychotics are of particular concern. Of all antipsychotics, clozapine and olanzapine have the highest risk of weight gain (43, 46). Both schizophrenia and bipolar disorder are associated with an increased risk venous thromboembolism (VTE) in form of deep-vein thrombosis (DVT) or pulmonary embolism (PE). This increased risk of venous thromboembolism may be related to a higher risk of smoking and obesity in individuals with SMD. Immobilization, including lack of exercises, restraints and lower leg paralysis, and treatments with antipsychotics may constitute further risk factors for VTE (47, 48). Antipsychotics have also been implicated to increase the risk of VTE. Risk estimates range from 50% to 3-fold increased risk, depending on substance class (49, 50).

## Psychosocial Stress

Based on previous experience from accounts of the Spanish flu 1918–1919 and recent respiratory infection outbreaks such as the SARS outbreak in 2002–2003, there is reason for concern that psychiatric conditions and suicide rates may increase under the ongoing pandemic. Anxiety, depression and post-traumatic stress syndrome (PTSD) may all become more prevalent (51–53). A COVID-19 infection, or fear of it, or physical distancing and related income loss and unemployment would represent the types of stresses that could exacerbate severe mental illness (54–57). Added economic stress and particularly unemployment could then further elevate levels of severe mental illness and suicide (57–59). Individuals with SMD may be particularly vulnerable because SMD in itself and its co-morbidities are likely associated with lower socioeconomic status (57). This would be consistent with findings from the US that COVID-19 mortality is more frequent in lower socioeconomic ethnic minority groups (60, 61). The impact of socioeconomic status and ethnicity on the risk of COVID-19 infection and mortality in individuals with SMD requires further study. As demonstrated, individuals with SMD are more vulnerable to stress, and in turn, stress may make affected individuals more vulnerable to a COVID-19 infection. Susceptibility to respiratory infections has been shown to increase under stressful conditions (62, 63). Possibly, prolonged stressors result in glucocorticoid receptor resistance, which then alters the local pro-inflammatory cytokine response to an infectious agent (64). In summary, a circularity could ensue between SMD, comorbid conditions, economic disruption and COVID-19 infection, where all factors precipitate or amplify each other. Similar circularities have been observed previously, for instance during the Great Depression in the US in the 1930 (57).

### Strengths

The major strength of this study is its large sample-size and representativeness with register data covering the entire Swedish population aged 20 years and older. Therefore, there is no scope for selection bias. Individuals fell into one group (SMD) or the other group (reference population); no further exclusion criteria were warranted. The summary data was prepared independently from the research group by a statistician at the Swedish Board of Health and Welfare. Hence, the scope for observation bias was minimized. A further strength lies in the accuracy of Swedish register data. For instance, the Swedish Cause of Death Register covers more than 99% of all deaths. Obviously, despite covering the whole Swedish population, our findings may not automatically generalizable to all population groups within Sweden, or as it matters to populations outside of Sweden. However, our findings are in line with other population-based studies from the US and the UK who found excess of COVID-19 associated mortality of similar magnitude.

### Limitations

Our data covered the early months of the COVID-19 outbreak in Sweden. In these early months, numbers of deaths were much higher with an average of 54 deaths per day during our observation period. The numbers of deaths then substantially dropped off from the beginning of August and the mortality curve has flattened out to an average of 4 deaths per day in October. At the time of writing at the end of November, the numbers of death have risen again, but nowhere near to previous levels and not in keeping the number of infections (65). Equally it remains unclear how this would affect the odds of COVID-19 associated death in individuals with SMD. In this preliminary report based on register data, it was not possible to distinguish if any increased odds of death due to COVID-19 in individuals with SMD was due to an increased risk of contracting the SARS-CoV-2 virus, or due to an increased risk of a severe course of COVID-19 illness resulting in death.

Individuals were classified as having severe mental disorders if they were diagnosed twice between 1998 and 2019. Theoretically, an individual diagnosed early on, for instance in 1998, but then remained symptom-free could be entered in the SMD category. This could have led to misclassification. However, this would most likely have resulted in an underestimation rather than an overestimation of the odds of COVID-19 associated death in individuals with SMD. Such a misclassification would therefore not invalidate our results. The scope for misclassification was further reduced by the requirement of having two such diagnoses registered. Besides, bipolar disorder and schizophrenia are both chronic disorders, often of life-long duration. Symptomatic remission may not be equated with functional recovery and residual symptoms may persist (66, 67). We intend to extend our analysis, which is currently based on summary data, with individual level data as soon as possible.

In order to maximize power, we amalgamated bipolar and psychotic disorders into SMD as one exposure category. Several other examples of epidemiological studies exist, where mood and psychotic disorders are amalgamated in similar ways (3, 22, 48, 68–70). Here, we combined psychotic and bipolar disorders into one category because the prevalence for somatic comorbidities and excess mortality are similar for both conditions (1, 2). We did not include severe depression in our SMD variable, since severe depression is a more heterogeneous group. For this group, based on register data alone, it can be difficult to establish whether depression is the cause or consequence of somatic comorbidity. However, based on the available literature, we have reason to believe that including individuals with depression into our study would not have substantially altered the results.

The US study examining the association between mental disorder and death associated with COVID-19 infection did not only find a 2-fold death rate in patients with mental disorder as compared with patients without any mental disorder. The death rate was also approximately double for patients with depression only (8.2%) (18). The UK study exploring the association between pre-existing comorbidities, COVID-19 infection and death found 80% increased odds of dying with a pre-existing diagnosis of depression (20). As pointed out previously, these figures are very similar to our own. Including the ICD code F30, manic episode, could lead to an inclusion of individuals with one single hypomanic episode under the ICD sub-category of F30.0. However, as each individual required at least two registered diagnosis, the scope of including such individuals is virtually non-existent.

Another source of misclassification could arise if individuals with SMD were less likely to be tested for COVID-19 than the rest of the population. This again would lead to an underestimation rather than an overestimation of COVID-19 associated mortality in individuals with SMD. In the beginning of the pandemic, testing was not ubiquitously available. Thus, diagnoses were also made clinically. This is reflected by the provisional ICD code U07.2. Thus, there was scope for misclassification due to false positive COVID-19 diagnoses. In both groups, about 90% of diagnoses were confirmed by laboratory testing (U07.1). Hence, the scope for misclassification was comparably low in both groups. As acknowledged earlier in this discussion, not every death associated with COVID-19 infection may have been caused by COVID-19 infection (24, 25). Distinguishing between causation and association may have been particularly difficult in the early months of the COVID-19 outbreak. However, this would have affected cases and controls in the same way without any impact on the OR.

As pointed out previously, for this exploratory analysis, we had to rely on summary data. Summary data are much less detailed than individual level data. However, we decided to report our summary data at this point to alert clinicians to this new risk group. We intend to conduct further analyses with individual level data as soon as possible. With individual level data it will be possible to adjust for baseline variables, such as age and sex, specific mental disorder (psychotic vs. bipolar disorder), psychotropic drugs used, residence (urban vs. rural), and other variables of potential importance as outlined in our discussion. The association between COVID-19 associated mortality and SMD involves most likely a large quantity of biological and psychosocial factors. Some of these will be confounders, but others may actually lie as mediators on exposure-outcome causal pathways. Thus, even though our finding that SMD doubles the odds of COVID-19 associated death is crude and preliminary, it is nevertheless noteworthy. We intend to expand our analysis on individual data level and more detailed stratification and adjustment for confounders and mediators next year, when more longitudinal data is available. To further study the impact of SMD on the risk of COVID-19 infection or associated deaths, both register and clinical studies are needed. Register studies have the advantage of large sample sizes, but clinical information is limited. Clinical studies will be smaller, but risk factors can be explored in more detail.

It could be argued that it would be more informative to wait until more longitudinal data was available. However, our time frame is in keeping with the time frame chosen in the US studies reporting on mental disorders and SUD (18, 19). Our time frame is substantially longer than the time frame of the UK study reporting on depression (20). At the same time individuals with SMD may be particularly vulnerable at the beginning of a pandemic when health care resources are redirected toward acute somatic care. Finally, although a vaccine is not yet available, decisions about its allocation are made now. The Public Health Agency of Sweden states that those most in need of help to prevent serious illness from COVID-19 infection will be given top priority and which groups are given priority will be determined by the state of knowledge available when the vaccine arrives (71). Therefore, waiting for more longitudinal data would deprive individuals with SMD from the opportunity to be considered a risk group meriting priority (72).

## Conclusions

Our preliminary results suggest that individuals with SMD may be a further group at increased risk of COVID-19 associated death. It is important that clinicians are alerted to this new risk group. This increased mortality can be associated with a higher prevalence of somatic morbidity and life-style related factors. In regard to comorbidities, future studies should explore the potential confounding or mediation role in the relationship between SMD and COVID-19 associated deaths. Such clarification will help to enabling clinicians to provide the best physical and mental health care tailored to special requirements of this risk group.

## Data Availability Statement

The raw data supporting the conclusions of this article will be made available by the authors, without undue reservation.

## Ethics Statement

The studies involving human participants were reviewed and approved by Swedish Ethical Review Authority (DNR 2020-02759). Written informed consent for participation was not required for this study in accordance with the national legislation and the institutional requirements.

## Author Contributions

MM, UW, MB, LÖ, and MW: conception and design of the work and revising and providing the final approval of the work. MM, UW, and LÖ: acquisition and analysis of data. UW, MM, and MB: drafting the work. All authors contributed to the article and approved the submitted version.

## Conflict of Interest

UW has received funding for educational activities on behalf of Norrbotten Region (Masterclass Psychiatry Programme 2014–2018 and EAPM 2016, Luleå, Sweden): Astra Zeneca, Eli Lilly, Janssen, Novartis, Otsuka/Lundbeck, Servier, Shire, and Sunovion. The remaining authors declare that the research was conducted in the absence of any commercial or financial relationships that could be construed as a potential conflict of interest.
