# Supplementary material for: Death Associated With Coronavirus (COVID-19) Infection in Individuals With Severe Mental Disorders in Sweden During the Early Months of the Outbreak—An Exploratory Cross-Sectional Analysis of a Population-Based Register Study
Source: Front Psychiatry. 2021 Jan 8;11:609579. doi: 10.3389/fpsyt.2020.609579 (PMC7819873; doi:10.3389/fpsyt.2020.609579)
Supplement: Supplementary file 1 [file Table_1.doc]

**Appendix 1: STROBE Statement - checklist for our study (Maripuu M, Bendix M, Öhlund L, Widerström M, Werneke U: Death associated with coronavirus (COVID-19) infection in individuals with severe mental disorders in Sweden during the early months of the outbreak – an exploratory cross-sectional analysis of a population-based register study)**

| **STROBE requirement** | **#** | **Our study** |
| --- | --- | --- |
| *Title and abstract* | 1 |  |
| *(a)* Indicate the study’s design with a commonly used term in the title and abstract |  | *(a)*  Title: Study design included in title  *“Death associated with coronavirus (COVID-19) infection in individuals with severe mental disorders in Sweden during the early months of the outbreak – a exploratory cross-sectional analysis of a population-based register study”*  Abstract: Study design included in abstract  *“Methods: Exploratory analysis with a cross-sectional design in the framework of a population-based register study covering the entire Swedish population. The Swedish Board for Health and Welfare (Socialstyrelsen) provided anonymised tabulated summary data for further analysis. We compared numbers of COVID-19 associated death in individuals with SMD (cases) and without SMD (controls).”* |
| *(b)* Provide in the abstract an informative and balanced summary of what was done and what was found |  | *(b)* Structured abstract provided. |
| *Introduction* |  |  |
| Background/rationale: Explain the scientific background and rationale for the investigations being reported | 2 | Background outlined in introduction. |
| Objectives:  State specific objectives, including any pre-specified hypotheses | 3 | Objective clearly stated in text  *“To assess the risk of death associated with COVID-19 infection (COVID-19 associated death) in individuals with SMD. We tested the following hypothesis: Individuals with SMD have a higher risk of COVID-19 associated death than individuals without SMD (reference population).* “ |
| *Methods* |  |  |
| Study design:  Present key elements of the study design early in the paper | 4 | Described  *“Key elements of the study included in the method: study design, data sources, sample, variable definitions (outcomes and exposures) statistical analysis.”* |
| Setting:  Describe the setting, locations, and relevant dates, including periods of recruitment, exposure, follow-up, and data collection | 5 | Described in study design and data sources  “*We included the whole Swedish population of at least 20 years of age by 31 Dec 2019. We defined individuals with a diagnosis of SMD as cases and all other individuals as controls. As the sample covered the whole adult Swedish population, all individuals fell either into the category “SMD” or the category “without SMD”. Therefore, there were no other exclusion criteria other than age < 20 years.”…*  *…”In Sweden, the first confirmed case of COVID-19 infection was reported on 31^st^ January 2020.^10^ The first COVID-19 related death was reported on 11^th^ March 2020.^11^ Our outcome was COVID-19 associated* *death, registered as such by the Swedish Board for Health and Welfare. We included all COVID-19 associated deaths occurring over a three-month period, from 11 March 2020 until 23 June 2020.*  *The main exposure was SMD. We included bipolar or psychotic disorders with ICD-10 codes F20, F22, F25, F30 or F31 into the SMD variable. Individuals were included in the SMD category when there were at least two registered diagnoses between 1998 and 2019.”* |
| Participants:  *(a)* Give the eligibility criteria, and the sources and methods of case ascertainment and control selection. Give the rationale for the choice of cases and controls  (*b*) For matched studies, give matching criteria and the number of controls per case | 6 | *(a)* Sample described.  *“We included the whole Swedish population of at least 20 years of age by 31 Dec 2019. We defined individuals with a diagnosis of SMD as cases and all other individuals as controls. As the sample covered the whole adult Swedish population, all individuals fell either into the category “SMD” or the category “without SMD”. Therefore, there were no other exclusion criteria other than age < 20 years. The cut-off of 20 years was chosen, because we used multiples of ten years to stratify our data. 18-20 years was left out, because of the short age span and expected low risk of COVID-19 associated death in young people.”*  (b) N/A |
| Variables:  Clearly define all outcomes, exposures, predictors, potential confounders, and effect modifiers. Give diagnostic criteria, if applicable | 7 | Definitions of all variables, outcomes and exposures, clearly defined in text. |
| Data sources  /measurement:  For each variable of interest, give sources of data and details of methods of assessment (measurement). Describe comparability of assessment methods if there is more than one group | 8 | Source:  Described in text  *“The Swedish National Patient Register is based on diagnoses for both in and outpatient care in specialised medicine (secondary care). Diagnoses from general practitioners (primary care) are not included in this register. The Swedish National Death Register includes all Swedish persons that have died. The cause of death is established in either primary or secondary care, depending on where the death has occurred. The Swedish Prescribed Drug Register contains data on treatments that were dispensed at a pharmacy.”* |
| Bias: Describe any efforts to address potential sources of bias | 9 | Addressed in discussion  *“The study is representative with register data covering the entire Swedish population aged 20 years and older. Therefore, there is no scope for selection bias. Individuals fell into one group (SMD) or the other group (reference population); no further exclusion criteria were warranted. The summary data was prepared independently from the research group by a statistician at the Swedish Board of Health and Welfare. Hence, the scope for observation bias was minimised.”* |
| Study Size:  Explain how the study size was arrived at | 10 | Described in study design and sample |
| Quantitative variables: Explain how quantitative variables were handled in the analyses. If applicable, describe which groupings were chosen and why | 11 | Described, cf. Statistical methods |
| Statistical methods: *a)* Describe all statistical methods, including those used to control for confounding  *(b)* Describe any methods used to examine subgroups and interactions  (*c)* Explain how missing data were addressed  *(d)* If applicable, explain how matching of cases and controls was addressed  *(e)* Describe any sensitivity analyses | 12 | *(a)* Described in methods and discussed in limitations  *“For this exploratory analysis, we had to rely on summary data. Summary data are much less detailed than individual level data. However, we decided to report our summary data at this point to alert clinicians to this new risk group. We intend to conduct further analyses with individual level data as soon as possible. We intend to conduct further analyses with individual level data as soon as possible. With individual level data it will be possible to adjust for baseline variables, such as age and sex, specific mental disorder (psychotic versus bipolar disorder), psychotropic drugs used, residence (urban versus rural), and other variables of potential importance as outlined in our discussion.”*  *(b)* Main stratification according to SMD, further stratification according to age groups and risk factors  *(c)* Methods to address missing data discussed  *“The number of deaths was available for all age groups. However, for some risk factor categories, the number of deaths had been withheld due to confidentiality reasons. For summary estimates of risk factors in the whole age group, we set missing data to 0.”*  Cf. also methods to control for bias.  *(d)* N/A  *(e)* N/A |
| *Results* |  |  |
| Participants:  *(a)* Report numbers of individuals at each stage of study—eg numbers potentially eligible, examined for eligibility, confirmed eligible, included in the study, completing follow-up, and analyzed  *(b)* Give reasons for non-participation at each stage  *(c)* Consider use of a flow diagram | 13 | *(a)* Described in text and flow chart.  *(b)* N/A. The outcome and the main exposure SMD were available for all participants.    *(c)* A flow diagram would not provide further clarification: The study covered the entire Swedish population aged 20 years and older. *“Individuals fell into one group (SMD) or the other group (reference population); no further exclusion criteria were warranted.”* |
| Descriptive data:  *(a)* Give characteristics of study participants (e.g. demographic, clinical, social) and information on exposures and potential confounders  *(b)* Indicate number of participants with missing data for each variable of interest | 14 | *(a)* Described in sample  *(b)* Missing data addressed  *“The number of deaths was available for all age groups. However, for some risk factor categories, the number of deaths had been withheld due to confidentiality reasons. For summary estimates of risk factors in the whole age group, we set missing data to 0.”* |
| Outcome data:  Report numbers in each exposure category, or summary measures of exposure | 15 | Outcome data presented in text and tables 1 and 2 |
| Main results  *(a)* Give unadjusted estimates and, if applicable, confounder-adjusted estimates and their precision (eg, 95% confidence interval). Make clear which confounders were adjusted for and why they were included  *(b)* Report category boundaries when continuous variables were categorized  *(c)* If relevant, consider translating estimates of relative risk into absolute risk for a meaningful time period | 16 | (a) *Cf. result and Tables 1 and 2*  Variable selection and potential other confounding factors discussed  *“For our study, we chose four risk factors thought to be more prevalent in individuals with SMD.^21^ We chose these risk factors during the set-up of the study. At the time, evidence regarding risk factors was only emerging. Therefore, we made an informed guess that these four risk factors could affect the risk of COVID-19 associated mortality”…*  *…”For this exploratory analysis, we had to rely on summary data. Summary data are much less detailed than individual level data. However, we decided to report our summary data at this point to alert clinicians to this new risk group. possible. With individual level data it will be possible to adjust for baseline variables, such as age and sex, specific mental disorder (psychotic versus bipolar disorder), psychotropic drugs used, residence (urban versus rural), and other variables of potential importance as outlined in our discussion.”*  *(b)* Category boundaries for exposure variables described  *(c) cf. (a)* |
| Other analysis:  Report other analyses done—e.g. analyses of subgroups and interactions, and sensitivity analyses | 17 | Main stratification according to SMD, further stratification according to age groups and risk factors |
| *Discussion* |  |  |
| Key results:  Summarize key results with reference to study objectives | 18 | Done |
| Limitations:  Discuss limitations of the study, taking into account sources of potential bias or imprecision. Discuss both direction and magnitude of any potential bias | 19 | Limitation discussed in terms of variable definitions, use of summary data instead of data at individual level, choice of potential confounder variables, limited scope to adjust for confounders, capture of outcome |
| Interpretation:  Give a cautious overall interpretation of results considering objectives, limitations, multiplicity of analyses, results from similar studies, and other relevant evidence | 20 | Done |
| Generalisability:  Discuss the generalizability (external validity) of the study results | 21 | Discussed  *“The major strength of this study is its large sample-size in a naturalistic setting. The study is representative with register data covering the entire Swedish population aged 20 years and older. Therefore, there is no scope for selection bias. Individuals fell into one group (SMD) or the other group (reference population).”*  Use of SMD as an outcome variable discussed  *“In order to maximise power, we amalgamated bipolar and psychotic disorders into SMD as one exposure category. Several other examples of epidemiological studies exist, where mood and psychotic disorders are amalgamated in similar ways.^3,22,48,68-70^”*  Use of summary data instead of individual level data discussed  *“For this exploratory analysis, we had to rely on summary data. Summary data are much less detailed than individual level data. However, we decided to report our summary data at this point to alert clinicians to this new risk group. We intend to conduct further analyses with individual level data as soon as possible. With individual level data it will be possible to adjust for baseline variables, such as age and sex, specific mental disorder (psychotic versus bipolar disorder), psychotropic drugs used, residence (urban versus rural), and other variables of potential importance as outlined in our discussion”* |
| Funding:  Give the source of funding and the role of the funders for the present study and, if applicable, for the original study on which the present article is based | 22 | Funding sources given: This work was supported by the County Council of Jämtland/Härjedalen and the Department of Clinical Sciences, Umeå University and the Department of Psychiatry, Sunderby Hospital, Region Norrbotten, Sweden.  Conflict to interest statement for all authors included in manuscript. |

# Source: <http://www.strobe-statement.org/>. Accessed 14 September 2020
